# RNA-Binding Proteins as Regulators of Internal Initiation of Viral mRNA Translation

**DOI:** 10.3390/v14020188

**Published:** 2022-01-19

**Authors:** Brenda López-Ulloa, Yazmín Fuentes, Magdalena S. Pizarro-Ortega, Marcelo López-Lastra

**Affiliations:** Laboratorio de Virología Molecular, Instituto Milenio de Inmunología e Inmunoterapia, Departamento de Enfermedades Infecciosas e Inmunología Pediátrica, Centro de Investigaciones Médicas, Escuela de Medicina, Pontificia Universidad Católica de Chile, Marcoleta 391, Santiago 8330034, Chile; bplopez@uc.cl (B.L.-U.); yaz.bqa@gmail.com (Y.F.); mfpizarro@uc.cl (M.S.P.-O.)

**Keywords:** internal ribosome entry site, IRES, RNA-binding protein, RBP, IRES-transacting factor, ITAF

## Abstract

Viruses are obligate intracellular parasites that depend on the host’s protein synthesis machinery for translating their mRNAs. The viral mRNA (vRNA) competes with the host mRNA to recruit the translational machinery, including ribosomes, tRNAs, and the limited eukaryotic translation initiation factor (eIFs) pool. Many viruses utilize non-canonical strategies such as targeting host eIFs and RNA elements known as internal ribosome entry sites (IRESs) to reprogram cellular gene expression, ensuring preferential translation of vRNAs. In this review, we discuss vRNA IRES-mediated translation initiation, highlighting the role of RNA-binding proteins (RBPs), other than the canonical translation initiation factors, in regulating their activity.

## 1. Overview

Viruses depend on the host cell for their replication. This dependency is evident during protein synthesis as viruses, except for giant viruses [1], lack any component of the translational apparatus. Hence, the translation of viral messenger RNA (vRNA) exclusively relies on the cellular translational machinery. Therefore, vRNAs compete with cellular messenger RNAs (mRNAs) for the machinery required for protein synthesis. Success in this competition ensures the host’s translational machinery’s reprogramming towards preferentially synthesizing viral proteins (for reviews, see [2,3,4,5]). Many vRNAs have evolved to exploit a range of unconventional mechanisms for efficient 40S ribosomal subunit recruitment to guarantee success in this asymmetrical competition, enabling vRNAs to outcompete their numerous cellular counterparts at the stage of translation initiation [2,4,6,7,8]. Various strategies used by viruses to usurp the host’s translational machinery rely on their ability to modify the cellular environment during their replication, repurposing the function of many cellular RNA-binding proteins (RBP) to favor the translation of vRNAs [2,4,6,7,8]. This review briefly describes the canonical cap-dependent mechanism of translation initiation used by most cellular mRNAs. As this stage in protein synthesis has been extensively reviewed [9,10,11,12,13,14], we omit to discuss alternative non-canonical cap-dependent mechanisms used by some eukaryotic mRNAs [9,11,15,16,17,18,19,20]. The review describes internal ribosome entry site (IRES)-mediated translation initiation of vRNAs as a viral strategy to outcompete cellular mRNAs in recruiting the translational machinery during infection. We highlight the role of a particular subset of RBPs, known as IRES-transacting factors (ITAFs), in regulating the activity of viral IRES via protein-RNA and protein–protein interaction. Finally, we discuss the impact of internal initiation on viral tropism.

## 2. Cap-Dependent Translation Initiation

**Figure 1:**
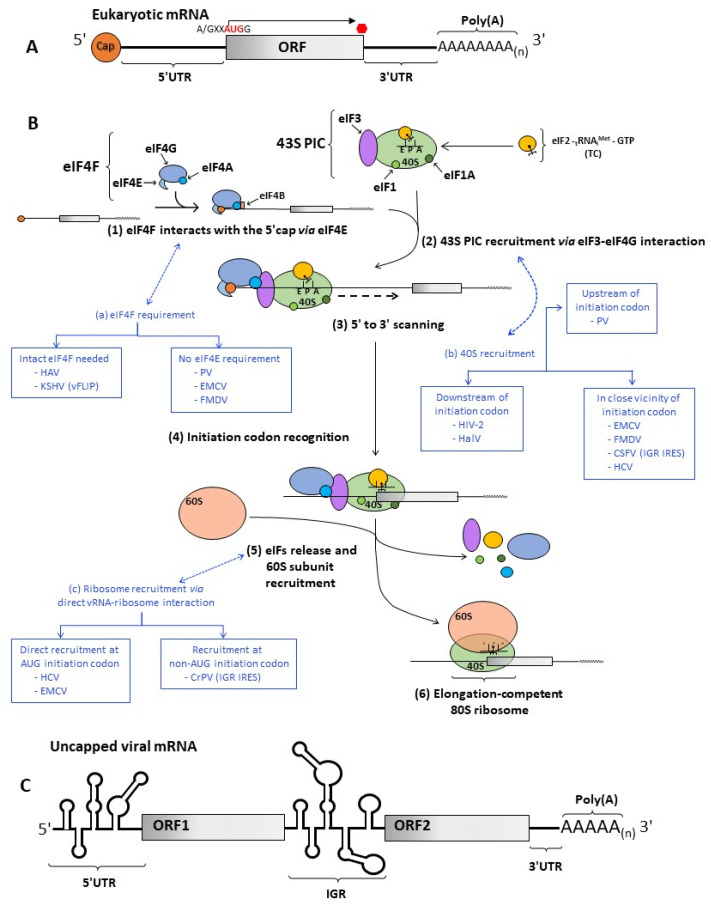
Cap-dependent translation initiation and viral strategies for ribosomal recruitment. (**A**) Schematic representation of a eukaryotic mRNA, including the 5′ cap structure, the 5′ untranslated region (UTR), the open reading frame (ORF), followed by the 3′ UTR and the Poly(A) at the 3′ end. The initiation codon (red) is also highlighted as part of the optimal context. (**B**) Schematic representation of canonical cap-dependent translation initiation summarized in 6 steps. Circularization of the mRNA is omitted. The eIF4F complex, 43S-preinitiation complex (PIC), and ternary complex (TC) are shown. The presence of the cap structure at the 5′ end of the mRNA allows for the recruitment of the eIF4F complex (1) and the 43S PIC (2). After these complexes scan the mRNA, they travel in an ATP-dependent, 5′ to 3′ direction (3). During scanning, eIF4A, in association with cofactor eIF4B, unwinds the secondary structure of the 5′ UTR. Scanning ends when the initiation codon is recognized (4). The factor eIF1 and eIF1A help avoid non-initiation codon interaction with the tRNA^Met^_i_ placed in the P-site of the 40S subunit. Codon:anticodon interaction allows for the hydrolysis of eIF2-GTP and triggers the release of several factors, contributing to the recruitment of the 60S subunit (5) and the release of the remaining initiation factors, leading to the assembly of an elongation-competent 80S ribosome (6). Some viral strategies to recruit the ribosome by IRES are shown in blue: (a) whether intact eIF4F complex is needed; (b) regarding the position of the 40S subunit recruitment; and (c) whether the vRNA directly interacts with the ribosome. (**C**) Schematic representation of *Dicistroviridae* RNA, highlighting the IRES present in the 5′ UTR and the intergenic region (IGR).

The eukaryotic mRNA features a 5′ end modification known as the “cap”, a 7-methylguanosine linked, via a 5′ to 5′ triphosphate bridge, to the first transcribed nucleotide (m7GpppN) [21] and, except for histone encoding transcripts [22], also harbors a 3′ poly(A) tail [23] (Figure 1A). The translation of eukaryotic mRNAs comprises the initiation, elongation, termination, and ribosome recycling steps [9,11,14]. The control of eukaryotic mRNA translation is mainly, but not uniquely, exerted at the initiation step (Figure 1B), where the 5′ cap is recognized by the eukaryotic initiation factor (eIF) 4F, a heterotrimeric complex comprising eIF4E, eIF4A, and eIF4G [9]. The cap-binding protein, eIF4E, recognizes the cap, eIF4A, which is an ATP-dependent RNA helicase; it unwinds RNA structures present within the 5′ untranslated region (UTR) of the mRNA, and eIF4G acts as a scaffold, bridging eIF4E, eIF4A, and the 40S ribosomal subunit (via eIF3) [9]. EIF4G also interacts with the poly(A)-binding protein (PABP) that covers the poly(A) tail, noncovalently circularizing the mRNA in a “closed-loop” conformation. This non-covalent interaction between the 5′ and 3′ end of the mRNA promotes translation initiation, translation re-initiation through ribosome recycling and enhances mRNA stability [23,24,25,26]. In mammals, the 40S ribosomal subunit is recruited to the mRNAs as part of the 43S pre-initiation complex (PIC) that also includes eIF1, eIF1A, eIF5, the ternary complex (TC), eIF2-GTP-Methionine-initiator tRNA (tRNAi), and the multisubunit complex eIF3, through the association of eIF4G and eIF3. The binding of eIF1 to the 40S subunit sterically blocks 60S association, and eIF1A blocks the site for elongation tRNA binding [27]. EIF1A and eIF1 favor the scanning-permissive 40S open conformation [28]. Upon recruitment and formation of the 48S complex, the 40S ribosomal subunit scans the mRNA 5′ UTR in a 5′ to 3′ direction in an ATP-dependent fashion, probing for the codon (mRNA):anticodon (tRNAi) interaction. During scanning, the unwinding of RNA secondary structures is managed mainly by eIF4A, eIF4AI (DDX2A), eIF4AII (DDX2B), and cofactors eIF4B or eIF4H, which couples ATP-hydrolysis and RNA unwinding [29]. In the case of highly structured 5′ UTRs, where the RNA unwinding activity of eIF4A-eIF4B (or eIF4H) might be insufficient, auxiliary RNA helicases, including Ded1, DHX29, VAS, RHA (DHX9), can be recruited to the complex, assisting in the process of scanning [29]. Scanning continues until the initiation codon, generally, the first AUG triplet, in the adequate context (A/GXXAUGG; where X corresponds to any nucleotide), is encountered and recognized [12,13,14]. During scanning, eIF1A and eIF1 assist in dissociating aberrant codon-anticodon interactions by preventing the binding of the tRNAi to near-cognate initiation codons or initiation codons in a poor context [30,31]. The tRNAi is brought to the PIC by eIF2, a GTPase composed of three (α, β, and γ) subunits. The GTPase activity of eIF2 requires the GTPase activating protein (GAP) eIF5. In the complex, eIF1 inhibits the function of eIF5, obstructing tRNAi accommodation within the peptidyl (P) site, ensuring, in this way, the fidelity of initiation [11,32,33]. The codon:anticodon base-pairing shifts the tRNAi from an unaccommodated P-OUT state to an accommodated P-IN state [34]. This conformational rearrangement triggers the release of eIF1 from the complex, switching the 40S ribosomal subunit to a closed scanning-incompetent conformation, halting scanning [11,13]. The codon:anticodon interaction and the 40S ribosomal subunit closed conformation are stabilized by eIF5, preventing eIF1 from rebinding the complex [35]. The absence of eIF1 promotes eIF5-mediated hydrolysis of the eIF2-bound GTP, leading to the release of eIF2-GDP and eIF5 from the complex [12,13,33], allowing eIF5B, a ribosome-dependent GTPase, to bind to the 40S subunit. Together eIF1A and eIF5B mediate the joining of the 60S ribosomal subunit, yielding the 80S ribosome [11,14,36,37]. Subsequently, eIF1A and eIF5B are released from the 80S ribosome [11,14,38]. In parallel, eIF2-GDP is recycled by exchanging its bound GDP for GTP with the assistance of eIF2B [39]. The step of translation initiation ends with an assembled elongation–competent 80S ribosome assembled onto the mRNA initiation codon with the tRNAi positioned at the ribosomal P site (Figure 1B).

## 3. IRES-Dependent Translation Initiation

The study of the monocistronic uncapped/polyadenylated vRNAs of poliovirus (PV; genus *Enterovirus*) and the encephalomyocarditis virus (EMCV; genus *Cardiovirus*), both members of the Picornaviridae family of viruses, pioneered the discovery of IRES-mediated translation initiation [40,41,42]. IRESs were functionally identified by inserting the PV or the EMCV 5′ UTR into the intercistronic spacer of a bicistronic construct encoding two reporter proteins [41,42]. In the context of this artificial bicistronic mRNA, the second cistron’s expression documented the inserted sequence’s ability to promote internal initiation [41,42,43]. IRESs were then defined as structural RNA elements that enable internal recruitment of the ribosome independently from a 5′ cap. A later study placed the 5′ UTR of EMCV in a covalently closed circular RNA (cccRNA) and demonstrated its ability to drive translation in a 5′ and 3′ end independent fashion [44]. In addition, these studies showed that the IRESs did not require other regions of the viral RNA to promote translation [41,42,43,44], setting bicistronic mRNAs as the gold standard experimental system to identify IRESs [41,42,43,45]. It is noteworthy that bicistronic RNA experiments require rigorous controls, as alternative splicing events or cryptic promoter activity of the used DNA vector can yield unwanted monocistronic mRNAs for the second cistron, misleading conclusions [45]. When designing the bicistronic assay, experimental controls for ribosomal readthrough and shunting should also be considered [45]. Once discovered in PV and EMCV, IRESs were promptly identified in the vRNAs of other members of the *Picornaviridae* [46,47,48]. Translation driven by the PV, EMCV, and other picornaviral IRESs was stimulated by the presence of the poly(A) tail [49,50,51,52,53,54], suggesting that, although not essential for its function, 5′-3′ end RNA communication favors IRES-mediated translation initiation.

The discovery of IRESs in picornaviruses and the development of experimental strategies to functionally define and characterize cap-independent translation initiation (reviewed in [43,45]) paved the way for discovering IRESs in a wide variety of vRNAs from divergent viral families. It is noteworthy that no consensus primary sequence or RNA structure predicts an IRES [45,55,56,57]. Consequently, IRESs are only defined functionally and identified experimentally [45,55,56,57]. However, efforts are being made to develop bioinformatics tools to identify RNA elements that enable internal initiation [58,59,60,61,62,63,64]. Besides *Picornavirales*, to date, IRESs have been recognized in the vRNAs of *Flaviviridae* [65,66], *Retroviridae* [67,68], and *Herpesviridae* [69,70,71]. IRESs are also present in the vRNA of fungal [72], insect [73,74], and plant [75,76,77] viruses. Notwithstanding, IRESs are not restricted to vRNAs and have also been discovered in cellular mRNAs, emerging as important modulators of gene expression in several cellular processes [78,79,80,81,82,83,84,85]. The IRES present in the 5′ UTR of the mRNA encoding the immunoglobulin heavy-chain binding protein (BiP, also known as heat shock protein 70-5 (HSPA5), and Glucose-regulated protein 78 (Grp78)) was the first to be documented [86]. Since, several reports suggest that 10–15% of cellular mRNAs can initiate internally [57,79,82,87]. Several databases, including the IRESdb [88], IRESite [89,90], IRESbase [91], and the human IRES atlas [92], list viral and cellular IRESs.

Viral IRESs are, in general, highly structured RNA segments that, when compared, are highly diverse in nucleotide (nt) length, primary sequence, and secondary/tertiary structure [82,93,94,95,96]. For example, the Picornaviral IRESs are up to ~450 nts long, the Hepatitis C virus (HCV, *Flaviviridae*, genus *Hepacivirus*) IRES is ~332 nts in length [97], while the Zika virus (ZIKV, *Flaviviridae*, genus *Flavivirus*) IRES is ~107 nts in length [66]. The Dengue virus (DENV, *Flaviviridae*, genus *Flavivirus*) vRNA harbors the shortest viral IRES documented to date, being only ~96 nts in length [66]. IRESs may also vary in the position where they dock the 40S ribosomal subunit on the vRNA (Figure 1B) [82,94,96,98,99]. The IRES of PV recruits the 40S ribosomal subunit upstream of the initiation codon [98,100,101], followed by a 5′–3′ scanning towards the initiation codon. In contrast, the HCV IRES recruits the 40S ribosomal subunit directly over the initiation codon [97,102]. IRESs also vary in the number and position in which they are found within the vRNA. *Picornaviridae* vRNAs harbor a single IRES positioned in the 5′ UTR of the vRNAs [98,100,101]. It is noteworthy that the vRNA of the Cadicivirus (CDV, *Picornaviridae*, genus *Dicipivirus*) is the only known exception [103]. The CDV vRNA, which encodes two non-overlapping open reading frames (ORFs), has two IRESs: one in the vRNA’s 5′ UTR (~606 nts in length) and a second in the intergenic region (IGR; ~588 nts in length) [103]. The *Dicistroviridae* family of viruses (genera *Aparavirus*, *Cripavirus*, and *Triatovirus*) encompasses small non-enveloped viruses with vRNAs that harbor two non-overlapping large ORFs (ORF 1 and ORF2) (Figure 1C). Individual IRESs, the 5′ UTR IRES [73,104,105,106,107,108], and the IGR IRES [73,74,99,104,108], independently translate ORF1 and ORF2. Two structurally different IGR IRESs (type I and II) have been described in *Dicistroviridae* vRNAs [74]. The 5′ UTR and IGR IRESs have distinct properties and functional needs [74]. As an example, the 5′ UTR IRES in the vRNA of the cricket paralysis virus (CrPV, *Dicistroviridae*, genus *Cripavirus*) is ~352 nts long [107], while the IGR IRES (type I) is ~189 nts in length [104]. The 5′ UTR IRES mediates the translation of non-structural proteins encoded by ORF1, throughout the viral replication cycle, while the IGR IRES is active in the late phase of infection, driving the synthesis of structural proteins from ORF2 [109]. Little is known about the requirements of the factors for the CrPV 5′ UTR IRES to be active. However, its function depends on the ribosomal protein receptor for activated C-kinase 1 (RACK1) [110]. In contrast, the function of the CrPV IGR IRES is RACK1-independent [110]. The activity of both IRESs appears to be cell-type-dependent, the 5′ UTR IRES translation being weaker than IGR IRES translation in SL2 cells, but much stronger than the IGR IRES in *Aedes albopictus* mosquito cell lines [104,111]. The CrPV IGR IRES binds the 40S subunit with high affinity then recruits the 60S subunit; alternatively, the IGR IRES can directly recruit 80S ribosomes [73,74,99,112,113,114]. The CrPV IGR IRES does not require initiation factors, GTP, or even the tRNAi to assemble elongation-competent 80S ribosomes [73,74,108,112]. In the 40S/CrPV IGR IRES complex, the 40S P-site is occupied not by tRNAi but by the viral IRES that mimics the tRNAi, and the first decoded triplet is in the A-site of the ribosome [113,115]. Therefore, the N-terminal residue of the CrPV and other CrPV-like capsid protein precursors is either alanine (encoded by GCU or GCA) or glutamine (encoded by CAA), and not methionine [73,116]. It is noteworthy that the IGR IRESs remain the only IRESs that are independent of the Met-tRNAi for translation initiation. IGR IRESs are also found in the vRNA of the unclassified (+)ssRNA virus Halastavi arva virus (HalV, unclassified Riboviria) [117], isolated from the intestinal contents of freshwater carp (*Cyprinus carpio*) [118]. An IGR IRES, the vFLIP IRES, in the vRNA of the human gamma herpesvirus 8 (HHV8, *Herpesviridae*, genus *Rhadinovirus*), also known as the Kaposi’s sarcoma-associated herpesvirus (KSHV), has been reported [119]. It is noteworthy that, the exact position of the vFLIP IRES remains controversial as it might also include part of the upstream ORF, in which case, it would not be a strict IGR IRES [70,120,121]. Nonetheless, it is interesting to note that the vFLIP IRES is present in a capped-transcript of a DNA virus that synthesizes its transcripts in the cell nucleus [70,119,120,121,122]. Similarly, vRNAs of other DNA viruses, such as members of the Polyomaviridae, have also been reported to harbor an IRES [123]. The interplay between cap- and IRES-dependent translation initiation in these vRNA remains to be elucidated. Other capped-vRNAs that harbor IRESs are those from DENV, ZIKV, and retroviruses [66,68]. As an example of the latter, the capped-full-length vRNAs of the human immunodeficiency virus type 1 (HIV-1; *Retroviridae*, genus *Lentivirus*) [124,125], the simian immunodeficiency virus (SIV; *Retroviridae*, genus *Lentivirus*) [126], and the feline immunodeficiency virus (FIV; *Retroviridae*, genus *Lentivirus*) [127], also harbor an IRES within the 5′ UTR. The HIV-1 and SIV vRNA have a second IRES within the downstream group-specific antigen (Gag)-ORF [128,129,130,131]. Little is still known about the second IRES in the vRNA of other members of the *Retroviridae* [67,68]. The 5′ UTR IRES drives the synthesis of the main viral polyprotein Gag [124,126], while the ORF-IRES (Gag-IRES) drives the translation for a shorter isoform of Gag [128,130]. The capped HIV-2 RNA also harbors IRESs, but these are located within the vRNA’s ORF [131,132,133,134]. Therefore, in the case of the HIV-2 IRESs, the 40S ribosomal subunit is recruited downstream from the initiation codon [131,132,133]. The binding of the 40S ribosomal subunit to the HIV-2 or HIV-1 Gag-IRES requires only eIF3 [131]. In the case of HIV-2, upon recruitment, a retrograde scanning mechanism would position the subunit over the initiation codon [132]. A similar retrograde scanning strategy has been suggested for the HalV IRESs, which also recruits the 40S ribosomal subunit downstream of the initiation codon [135]. The molecular mechanisms driving initiation codon recognition by retrograde scanning remain unknown.

Viral IRESs also differ in the subset of host factors required to initiate translation (Figure 1B) [82,93,94,95,96]. In-Vitro reconstitution experiments showed the formation of 48S complexes on the EMCV, foot-and-mouth disease virus (FMDV; *Picornaviridae*, genus *Aphthovirus*), and PV IRESs, is ATP-dependent and requires the same initiation factors as the canonical cap-dependent initiation mechanism, except for eIF4E [82,96,136,137,138]. The eIF4F complex can be replaced by eIF4A and the carboxy-terminal fragment of eIF4G, lacking the eIF4E binding site [139]. The scanning factors eIF1 and eIF1A are required for the PV IRES activity as it recruits the 40S ribosome upstream of the initiation codon [82,96,138], but not for EMCV and FMDV IRES activities because they recruit the 40S ribosome at the immediate vicinity of the initiation codon; thus, scanning is not required. The PV, EMCV, and FMDV IRESs require ITAFs to function (as discussed below). In sharp contrast to other picornaviral IRESs, the hepatitis A virus (HAV) (*Picornaviridae*, genus *Hepatovirus*) IRES requires intact eIF4G to function [140,141]. However, even though HAV IRES requires the eIF4E-eIF4G interaction, the vRNA remains cap-independent, as the capping of monocistronic transcripts harboring the HAV IRES inhibits translation In Vitro [142]. Interestingly, the vFLIP IRES also requires the assembly of intact eIF4F onto the IRES to be functional [119]. The formation of the 40S/IRES complex over the HCV and Classical swine fever virus (CSFV, *Flaviviridae*, genus *Pestivirus*) is independent of any eIF [97,99]. In HCV mRNA, the 40S ribosomal subunit binds specifically to the HCV IRES without the requirement for any initiation factor, and the 40S P site is positioned in proximity of the initiation codon [102,143,144]. Subsequent addition of the TC to the 40S/HCV IRES complex is necessary and sufficient to form a functional 48S complex [102,144,145]. Significantly, initiation on the HCV IRES has no requirement for ATP or any factor associated with ATP hydrolysis and is resistant to the inhibition by dominant-negative eIF4A mutants [145]. Reports also suggest that the HCV IRES can directly hijack the host translational machinery while translation is ongoing by actively capturing translating 80S ribosomes [143,146].

For most vRNAs, IRES-mediated translation initiation is highly dependent on the structural integrity of the IRES [73,74,93,95,99,101,108,114,144,147,148]. RNA folding is a dynamic process involving short and long-range RNA–RNA interaction [149]. In accordance, mutations in stems negatively impact IRES activity, and compensatory mutations that reestablish the RNA secondary structure, but not its primary sequence, restore IRES-mediated translation [150,151,152]. Small deletions or insertions and even single nt substitutions can alter the folding of the IRES, abrogating or enhancing its function and directly impacting viral protein synthesis and viral replication [153,154,155,156]. For example, single substitutions in the PV IRES reduce vRNA translation, leading to PV attenuation [157,158,159]. Even in the CrPV IGR IRES, the mutation of two nts that disrupt the RNA structure is sufficient to abolish IRES activity [115,160]. However, the activity of some viral IRESs, such as the HIV-1 IRES [125,161,162,163], is not significantly affected by point mutations expected to disrupt RNA structural elements. Nonetheless, the deletion of RNA domains that constitute part of the core IRES negatively impacts its activity [125,161,164]. Moreover, not all viral IRESs are strictly dependent on highly structured RNA elements for their function. For example, the 148 nt IRES located upstream of the coat protein gene of crucifer-infecting tobamovirus (crTMV, *Virgaviridae*, genus *Tobamovirus*) is not structured, and its function is attributed to two polypurine (A-rich) sequences [165]. Likewise, the activity of the 5′-terminal Rhopalosiphum padi virus (RhPV, *Dicistroviridae*, genus *Cripavirus*) IRES relies on a non-structured RNA region, and 5′ and 3′ terminal deletions showed only minor effects on its activity [105,166].

Thus, viral IRESs behave as a complex RNA scaffold interacting with specific RBPs, the 40S ribosomal subunit, or with components of the canonical translational apparatus enabling translation initiation [167,168]. For example, the EMCV IRES directly interacts with eIF4G, an interaction enhanced by eIF4A [169,170]. Other picornaviral IRESs, such as the PV and FMDV IRES, also interact with eIF4G [138,153,171]. It is noteworthy that, in the absence of eIF4G/eIF4A, the EMCV IRES remains functional as the IRES alone can also recruit the 40S ribosomal subunit [172]. Reports indicate that most viral IRESs require a combination of eIFs and RBPs to be functional, suggesting that, rather than the RNA alone, it is the assembled ribonucleoprotein (RNP)-complex (the IRESome) that enables internal initiation [68,81,173].

## 4. IRES Classification

Despite the divergences in sequence, RNA structure, eIFs and ITAF requirements, and the molecular mechanisms involved in 40S recruitment, when they are from viruses from the same family and genus, IRESs tend to share features [14,82,93,95,96,99,174,175]. Viral IRESs have been classified into different classes or types based on similarities, such as: RNA structure resemblance, RNA structure complexity, the subset of eIFs needed for function, the need for ITAFs, direct or indirect interaction of the 40S ribosomal subunit with the vRNA, the place of docking of the 40S upon recruitment (upstream or in close vicinity of the initiation codon), and the requirement of 5′-3′ scanning. IRES classification has been extensively reviewed [14,82,93,95,96,99,175]. As a very general overview, we mention that the initial classification of IRESs considered only picornaviral IRESs. These IRESs were grouped into three types: type I, comprising the HAV IRES; type II, including the *Cardiovirus* and the *Aphthovirus*; and type III, the *Enterovirus* and *Rhinovirus* [46]. This classification was later rearranged into type I, which included the IRESs of Enteroviruses’ and Rhinoviruses’ IRESs, with PV becoming the prototype [98]. Type II included the IRESs found in the *Aphthovirus*, *Cardiovirus*, and *Parechovirus* genus vRNAs, with EMCV and FMDV IRESs as prototypes [98], and type III, included only the HAV IRES [98]. This classification successfully clustered most, but not all, picornaviral IRESs, leading to the later addition of IRES types IV and V [174,176,177]. Type IV cluster IRESs found in the vRNAs of Picornavirus genera *Aquamavirus*, Avihepatovirus, Megrivirus, Sapelovirus, Senecavirus, Teschovirus, *Tremovirus*, and the porcine kobuvirus (PKV), a member of the *Kobuvirus* genus. The minimal requirements for 48S assembly on the porcine teschovirus type 1 (*Picornaviridae*, genus *Teschovirus*) IRES, a member of the type IV IRESs, are the 40S ribosomal subunit and the TC [176]. However, the addition of eIF3 further enhances the formation of the 48S complex [176]. The less studied type V IRESs were identified in members of the *Kobuvirus*, *Salivirus*, and *Paraturdivirus* genera [177]. With the identification of IRES in the vRNA from other viral families, viral IRES classification has been constantly revisited as reviewed in [14,82,93,95,96,99,175]. Most classifications coincide with the criteria used to group viral IRESs and define four main groups. However, the different reports do not necessarily agree with the order given to the classified viral IRESs. For example, class I IRESs (Entero-/Rhinoviruses) and class II (Cardio-/Aphthovirus) in [14,82,93,96,175] correspond to groups IV and III, respectively, in [99]. The HAV IRES is alone in [82], but is grouped with the PV IRES in [99]. In addition, several known viral IRESs remain unclassified [82,96]. Independently of how viral IRESs are grouped, it is interesting to note that several highly diverse RNA structural elements have evolved in viruses to enable cap-independent recruitment of the host’s translational machinery.

## 5. Advantages of Having an IRES

During viral infections, the host triggers an antiviral response that includes, among other targets, blocking global translation to counteract viral protein synthesis [2,3,8]. However, successful viruses have evolved mechanisms to antagonize the host’s antiviral response at different stages, including vRNA translation, allowing viral protein synthesis to prevail even when global host protein synthesis is blocked in response to viral infection [2,3,8]. Some viruses target canonical mRNA translation as part of an aggressive takeover strategy, inducing the shutdown of host protein synthesis [4,7]. In this scenario, IRES-mediated translation initiation enables vRNAs to control the host’s translational machinery when cap-dependent translation is suppressed either by the cell, as part of its antiviral response, or by the virus itself, as part of its takeover strategy. For example, during infection, *Picornaviridae* deploy different strategies to suppress cap-dependent translation initiation. PV, coxsackievirus (CV, genus *Enterovirus*), and human rhinovirus (HRV, genus *Enterovirus*) encode the 2A proteases, while FMDV, the leader (L) protease (reviewed in [178,179]). These viral proteases cleave eIF4G, with the amino-terminal fragment keeping the eIF4E-binding site, while the carboxy-terminal portion retains the binding sites for eIF3 and eIF4A [7,180,181,182]. Cleavage of eIF4G shuts down cap-dependent translation initiation. However, the carboxy-terminal fragment of eIF4G is sufficient to withstand IRES-dependent translation initiation [181,183]. Interestingly, eIF4E stimulates 2A protease-induced cleavage of eIF4G [184,185]. In the case of PV, upon eIF4G cleavage, eIF4E is relocalized to the nucleus, further reducing cap-dependent translation initiation in infected cells [186]. During infection, enterovirus 71 (EV71, genus *Enterovirus*) induces microRNA (miRNA) 141 that targets eIF4E encoding mRNA [187]. Therefore, cap-dependent translation is reduced during active viral replication, while IRES-dependent mRNA remains functional, ensuring the efficient synthesis of the viral polyprotein. Furthermore, IRES-mediated translation is benefited by the virus-induced host’s protein shutdown, as evidenced when comparing the activity of the PV, HRV, and Echovirus 25 (ECHO, genus *Enterovirus*) IRESs in a neuronal cell line treated, or not treated, with the 2A protease [188]. PV, HRV, and ECHO IRES activity are barely detectable in neural cell lines [188]. However, in the presence of 2A protease PV, HRV, and ECHO IRES activity is rescued in neural cell lines [188]. A similar observation was made for the DENV IRES [189]. DENV IRES activity in HEK 293T cells was evident in cells expressing the HRV 2A protease [189]. Thus, in HEK 293T cells, DENV IRES is functional when cap-dependent translation initiation is inhibited. As eIF4G is a well-known target for the PV and HRV 2A proteases, it is tempting to conclude that eIF4G cleavage alone explains the increase in DENV IRES activity by reducing the competition with cap-dependent translation initiation. However, this was not directly evaluated [188,189]. Thus, the stimulation of DENV IRES activity could also be explained by a combined effect of the 2A proteases acting over the expression, localization, or stability of several proteins within the cells [178,181,182,190,191]. ZIKV NS2B-NS3 protease also targets eIF4GI, yet the impact of this cleavage on ZIKV IRES-mediated translation initiation has not been evaluated [192]. Retroviruses also encode a protease that targets eIF4GI, inhibiting cap-dependent translation initiation while favoring viral cap-independent translation initiation [68,193,194,195,196,197,198].

In contrast to PV and FMDV, EMCV does not cleave eIF4G; however, the virus reduces cellular cap-dependent translation initiation by inducing 4E-binding protein (4E-BP) dephosphorylation [199]. 4E-BPs and eIF4G occupy mutually exclusive binding sites on eIF4E [200,201,202]. However, the extent of 4E-BP phosphorylation modulates the 4E-BP–eIF4E interaction. When hypophosphorylated 4E-BPs strongly interact with eIF4E, displacing eIF4G, therefore hindering canonical eIF4E–eIF4G-dependent translation initiation and limiting eIF4F assembly [202]. In this scenario, EMCV IRES-dependent translation initiation prevails [199].

As mentioned above, DENV and ZIKV vRNAs possess a translationally functional 5′ cap-structure yet harbor an IRES [66,122]. Therefore, these vRNAs are expected to initiate translation using either a cap- or an IRES-dependent mechanism [66,189,203]. The IRES should enable these vRNAs to circumvent cellular regulatory mechanisms targeting cap-dependent translation initiation triggered during viral infection [66,189]. During infection, DENV and ZIKV remodel polysomes and suppress global translation; however, the production of viral proteins increases [204]. Consistent with this observation, vRNA is efficiently translated, while cellular mRNAs are not [204]. Early post-infection DENV induces the repression of host cell translation [204,205]. DENV replication causes cellular endoplasmic reticulum (ER) stress [206], leading to the oligomerization and activation of PERK that mediates the phosphorylation of eIF2α, resulting in the shutdown of global translation [207]. Reports suggest that DENV vRNA translation initiation transitions from a cap-dependent to a cap-independent mode during viral replication [66,189,203,205,208]. This conclusion is consistent with reports showing that DENV vRNA translation is resistant to the pharmacological suppression of cap-dependent translation initiation [203]. Furthermore, the DENV IRES activity is stimulated when cap-dependent translation initiation is suppressed [189]. Retroviruses are another example of capped vRNAs that harbor IRESs [67,68]. In the case of HIV-1, translation of the vRNA is most probably cap-dependent during the early stages of virus replication [198]. However, at later stages, cap-dependent translation initiation is suppressed by several virus-induced mechanisms, such as the blockage of the cell cycle in G2/M [209,210], viral-induced oxidative and osmotic stress [211,212], reducing eIF4E phosphorylation [210], increasing the amount of 4E-BP1 [210], and by viral protease-targeted eIF4G and PABP cleavage [194,196,197]. When cap-dependent translation is suppressed, the viral IRES enables HIV-1 protein synthesis [124,163,211,212,213,214]. Further validating this conclusion is the observation that HIV-1 IRES also allows vRNA translation in the presence of picornavirus proteases [124,213,214], confirming the ability of HIV-1 vRNA to translate under the conditions that induce a shutdown of cellular cap-dependent protein synthesis. Another example is the IRES present within the 5′ UTR of the capped FIV vRNA that is active under the stress conditions that hinder cap-dependent translation initiation [127]. It is noteworthy that, as previously reviewed [68,198,215], retroviral IRESs share features found in cellular IRESs rather than those exposed by IRESs from RNA viruses whose vRNAs lack a 5′ cap-structure.

## 6. IRES-Transacting Factors

**Figure 2:**
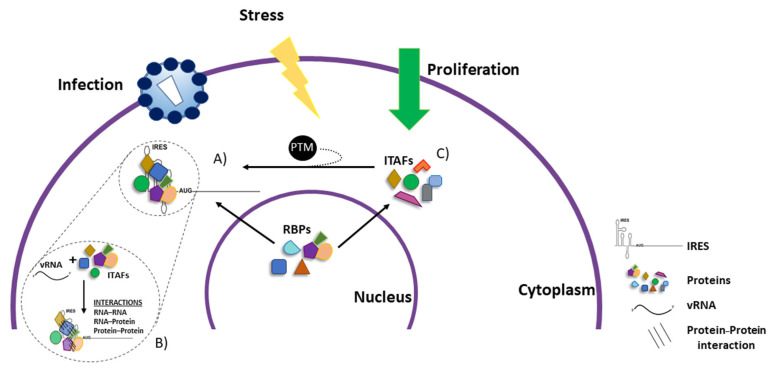
Nuclear and Cytoplasmic RBPs act as ITAFs. (**A**,**B**) IRES-transacting factors (ITAFs) are cytoplasmic or nucleus–cytoplasmic shuttling RNA-binding proteins (RBPs) [82,222]. (**C**) These ITAF–RNA and ITAF–ITAF interactions depend on cellular conditions such as stress, proliferation, or viral infection, which may trigger specific ITAF PTMs, that reduce or enhance the RBPs affinity for its target RNA. (**D**) ITAFs assist in RNA structuring by establishing multiple contacts with the same RNA molecules and other RBP [82,95,96,147,168,223]. Thus, RBPs induce RNA conformations or RNP complexes with differential affinities for the translation machinery.

**Figure 3:**
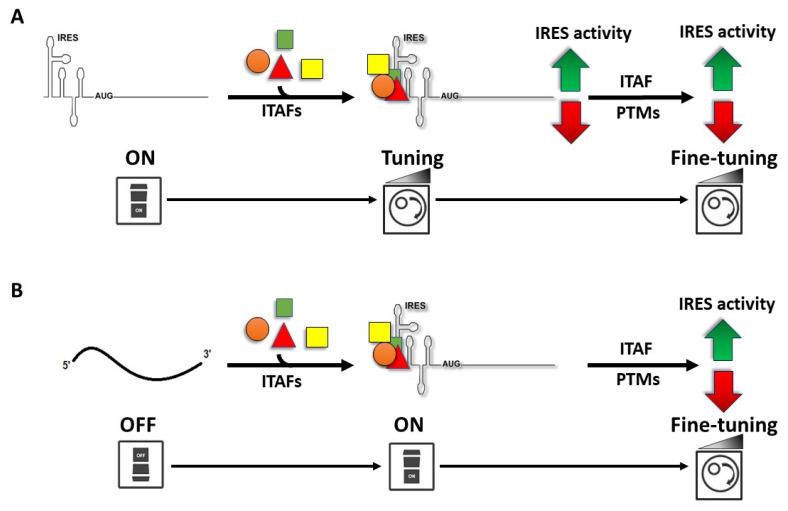
Model of IRES translational control mediated by RNA protein binding. (**A**) Some vRNAs do not require ITAFs to assemble (ON state). However, the association of different RBPs regulates the rate of translation initiation. ITAFs can also suffer PTMs, impacting their function over the IRES, further stimulating (green arrow) or reducing (red arrow) its activity [225]. (**B**) Some vRNAs (OFF state) require RBPs to assemble a functional IRES (ON state) [68,198]. ITAF PTMs can impact the ITAF localization, RNA affinity, or affinity for other RBPs impacting IRES function [81,225].

Early studies reported that the IRESs of EMCV, FMDV, and Theiler’s murine encephalomyelitis virus (TMEV, *Picornaviridae*, genus *Cardiovirus*), but not the IRESs of PV and HRV, were efficiently translated in rabbit reticulocyte lysate (RRL) [40,216,217]. However, in-vitro translation of the PV and HRV IRESs was enhanced when RRL were supplemented with HeLa cell extracts [40,216,217]. Similarly, the activity of the HAV IRES was stimulated in RRL supplemented with extracts prepared from mouse liver cells [218]. The HIV-1 IRES is not functional in RRL unless HeLa extracts made from G2/M arrested or unsynchronized cells are added [163]. Together these observations suggested that components of the cell extracts neutralized a translational inhibitor present in RRL, or that the RRL lacked at least one factor required for optimal translation that was provided by the cell extract. Later studies favored the second possibility, allowing the identification of cellular proteins that regulated IRES activity [147,168,219,220,221,222,223]. Thus, understanding how cell extract impacted IRES activity led to the discovery of ITAFs, RBPs that regulate (promote or inhibit) IRES activity (Figure 2) [82,222,223]. ITAFs possess chaperone activity and are thought to assist in RNA structuring [82,95,96,147,168]. ITAFs may establish multiple contacts within the same RNA molecule while interacting with other RBPs, stabilizing short and long-range RNA-RNA interactions (Figure 2) [82,95,168]. These ITAF–RNA and ITAF–ITAF interactions may depend upon the cellular environment that might impact or trigger specific ITAF post-translational modifications (PTMs), reducing or enhancing the RBPs affinity for its target RNA or other protein partners required for IRES activity (discussed below). By remodeling RNA structures via a combination of ITAF–RNA and ITAF–ITAF interactions, these RBPs may induce RNA conformations with a higher or lower affinity for the translation machinery, leading to an increase or a decrease in IRES-mediated translation initiation (Figure 3) [224].

The pyrimidine tract-binding protein (PTB, also known as heterogeneous nuclear ribonucleoprotein I (hnRNP I)) [216,226,227], and the La autoantigen [228,229,230], were the first identified ITAF for picornavirus IRESs. However, the list of ITAFs rapidly increased to include a significant number of well-known RBPs (Table 1). The list of RBPs that stimulated IRESs included: the poly(rC)-binding proteins 1 and 2 (PCBP1 and PCBP2, also known as hnRNP E1 and hnRNP E2) [231,232,233]; ErbB3-binding protein 1 (Ebp1, also known as ITAF_45_) [234,235]; the upstream of N-ras (Unr) [236,237,238]; the far upstream element (FUSE) binding protein 1(FBP-1) [239]; PTB Associated Splicing Factor (PSF) [240]; Elav-like protein HuR [241]; Argonaute 2 (Ago2) [241]; SRC associated in mitosis of 68 kDa (Sam68) [242]; heat-shock cognate protein 70 (Hsc70, also called HSPA8) and several of its family members including HSPA1 (or HSP72); the strictly inducible HSPA6 (or HSP70B′); and HSPA9 (or Glucose-regulated protein 75, GRP75) [243,244,245], among others (Table 1). Early reports suggested that proteins that interact with IRESs assemble large complexes consisting of several components [168,219,246,247], indicating that IRES function might require a combination of different ITAFs [234,236,248,249]. For example, PTB plus PCBP2 for the PV IRES [250]; PTB, Unr, and PCBP2 for the HRV IRES [236]; PTB plus Ebp1 for promoting the stable binding of eIF4G/4A to the FMDV IRES [234]; the splicing factor SRp20; and PCBP2 for the PV IRES [248,249]. The interplay between different ITAFs and their target IRES is still poorly understood, as some RBPs negatively modulate IRES-mediated translation initiation. For example, FBP-2 (also known as KH-type splicing regulatory protein KHSRP, KSRP, FUBP2, or P75) binds to the 5′ UTR of the EV71 IRES, negatively regulating vRNA translation [251]. It is noteworthy that during EV71 infection, the C-terminal region of FBP-2 is cleaved, and the processed protein now promotes EV71 IRES activity [252]. Therefore, throughout viral infection, ITAFs might be altered, and their function modified to benefit vRNA translation. EV71, PV, and HRV2 IRESs are also inhibited by hnRNP D (also known as AU-rich binding factor 1, AUF1) [253,254,255]. The hnRNP K and Gemin5 inhibit the FMDV IRES [256,257], while the double-stranded RNA-binding protein 76 (DRBP76, also known as NF90/NFAR-1, and ILF3), when heterodimerized with the nuclear factor of activated T cells, 45 kDa (NF45), selectively blocks HRV2 IRES-driven translation initiation in neuronal cells, but not in glioma cells [258].

As indicated above, the HCV IRES does not require ITAFs for 40S ribosomal subunit recruitment or 80S assembly [102]. However, HCV IRES-mediated translation is enhanced by the La autoantigen [272,306,307], HuR [224,293], Ago2 [303], Staufen1 [287], hnRNP L [275], hnRNP Q (also known as NSAP1) [276,277], and hnRNP D [271]. The case of PTB is less clear, as PTB binds the HCV IRES region [267,308,309], yet its impact on translation remains controversial [267,310,311,312,313,314]. In addition, Gemin5 binds the HCV IRES and inhibits its function [257,309]. PCBP1, PCBP2, and hnRNP A1 bind the HCV IRES, but their role in translation has not been fully evaluated [309,315,316]. Therefore, even though the HCV IRES does not require ITAFs for ribosome recruitment, these RBPs fine-tune viral protein by modulating, promoting or reducing, translation initiation (Figure 3).

The impact of a particular RBP on the function of an IRES cannot be predicted and has to be determined experimentally, because an ITAF that stimulates one viral IRES might act as a repressor for another. For example, HuR stimulates the EV71 and HCV IRESs [224,241,293], while it inhibits the activity of the HIV-1 IRES [224]. La autoantigen stimulates PV IRES activity [228,229,317], while it suppresses HAV IRES [274]. The molecular mechanism by which ITAFs promote or inhibit IRES-mediated translation initiation also varies. As mentioned above, ITAFs may act as chaperone proteins altering RNA folding and, consequently, IRES activity, or they may interfere or bridge the interaction between components of the translational machinery or other ITAFs and the vRNA [147,318]. Additionally, the same protein may use different mechanisms to modulate distinct IRES targets. In the case of the EMCV IRES, PTB binding to the core IRES limits the flexibility of the three-dimensional structure of the IRES, stabilizing its structure and promoting IRES activity [259]. However, for the PV IRES, PTB stimulates translation by inducing eIF4G to bind in the optimal position and orientation necessary to promote efficient internal ribosome entry [264]. The existence of different PTB mechanisms of regulation when associated with its target IRES is consistent with findings showing that different IRESs that use PTB as an ITAF exhibit sharp differences in the stringency of the PTB requirement for internal initiation [250,319,320]. In this sense, PTB is an absolute requirement for the entero- and rhinovirus IRESs [250]. The FMDV IRES also exhibits a strong dependency on PTB [319]. In contrast, PTB is stimulatory rather than essential for the function of the EMCV IRES, while the TMEV IRES is only marginally dependent on PTB [320]. Complexity is further added to the ITAF/IRES relationship when considering that most known ITAFs have different isoforms and paralogs, each of which might impact IRES activity differently. For example, PTB has three different isoforms resulting from alternative splicing, PTB1, PTB2, and PTB4 [321,322]. PTB1, PTB2, and PTB4 have four RNA recognition motifs (RRMs), RRM1 to RRM4, which mediate binding to pyrimidine-rich RNAs [322]. PTB2 and PTB4 variants differ from PTB1 only by an additional 19 or 26 amino acids between RRM2 and RRM3. In addition, PTB has two paralogs, the neural PTB (nPTB or brPTB), expressed at high levels in the adult brain, muscle, and testis, and ROD1 expressed in hematopoietic cells [322,323,324,325,326]. Despite their similarity, PTB1, PTB2, and PTB4 may exert different effects over the function of an IRES [268]. For example, PTB1 and PTB4, but not by PTB2, promote the mouse mammary tumor virus (MMTV; *Retroviridae*, genus *Betaretrovirus*) IRES activity [268]. PCBP (hnRNP E) has four isoforms [327,328], of which only PCBP1 (hnRNP E1) and PCBP2 (hnRNP E2) have been identified as ITAFs for the PV IRES [232,233]. Consistent with the need for this ITAF, the activity of the PV IRES is reduced in cell-free HeLa-based extracts depleted of PCBP2 [232]. The translational restriction associated with PCBP2 depletion is reverted only by the addition of PCBP2 and not by PCBP1, suggesting that one isoform cannot substitute the function of the other [232].

## 7. Most ITAFs Are Nucleus–Cytoplasmic Shuttling RNA-Binding Proteins

Several ITAFs for viral IRESs are nucleus–cytoplasmic shuttling RBP that possess multiple-RNA-binding domains (RBD), such as cold-shock domains (CSD), RNA-recognition motif (RRM), zinc fingers (ZF), double-stranded RBD (dsRBD), K-homology domain (KH), glycine-arginine-rich (GAR) domains (also named RGG boxes), as well as other less classical RBDs (Figure 2, Table 1) [297,329,330,331,332,333,334]. Interestingly, RBPs can also establish homo- and hetero- protein–protein interactions, assembling in such a way the IRESome, an RNP complex with translational activity [68,81,173]. Several ITAFs belong to the hnRNP family of proteins (for reviews, see [334,335,336,337,338,339,340,341,342]). HnRNPs were identified as proteins that bind to the primary transcripts of RNA polymerase II, heterogeneous nuclear RNAs (hnRNAs), or pre-mRNAs via specific RBDs, RRM, quasi-RRM, RGG box, glycine-rich auxiliary domains (RBD-Gly), and KH domains (Table 1) [337,338,339,340,341,342]. HnRNPs are abundant proteins with mainly nuclear localization that participate in numerous stages of mRNA metabolism, including splicing, RNA stabilization, and nucleus–cytoplasm transport. Most hnRNP proteins do, however, shuttle between the nucleus and the cytoplasm. The expected exceptions are hnRNP C and hnRNP U, which possess a nuclear retention sequence that reduced their transfer to the cytoplasm [343,344]. The hnRNP family comprises 20 major types ranging from 34 kDa (hnRNP A1) to 120 kDa (hnRNP U) [337,338,339,340,341,342]. Varied hnRNPs are recruited to transcripts in different relative amounts; hence, the protein composition of different RNP complexes is not fixed [345,346,347,348]. Furthermore, although widely expressed in cells, hnRNPs do vary in concentration between different tissues [349]. This variation can alter the conformation of the RNP complex when evaluated in different cellular backgrounds. The composition of the hnRNP complexes is transcript-specific, tissue-specific, and highly dynamic, being remodeled throughout mRNA metabolism and during the complex’s nucleocytoplasmic transport [337,338,339,340,341,342]. Thus, in the nucleus, mRNAs associate with a specific subset of RBP, many of which are hnRNPs, forming a transcript-dependent mRNP complex. This mRNP complex and its specific protein composition determine what has been called the “mRNP code”, which defines the fate of the mRNP complex in the cytoplasm [350].

Under normal conditions, most RBP/ITAFs reside in the cell nucleus. However, several viruses, including picornaviruses, subvert the host nucleocytoplasmic trafficking machinery to their benefit during infection (Figure 2) [334,351,352,353]. Different viruses, many of which rely on IRES-dependent translation initiation for the synthesis of their proteins, use different mechanisms to co-opt the cell’s nucleocytoplasmic trafficking machinery (for reviews, see [334,351,352,353]). For enteroviruses and rhinoviruses, 2A and 3C proteases are important for the process [191,354,355,356,357]. In aphthoviruses, the 3C protease is used [358], while cardioviruses need their L protein [359]. Nucleocytoplasmic protein relocalization during viral infection is not just a spillover event associated with viral infection as control nuclear proteins remain in the nucleus [351,352,353,360]. Proteins from the hnRNP family may, for example, associate with other proteins linked with pre-mRNA metabolism, such as members of the serine/arginine (SR)-rich protein family of splicing factors [336], relocalize as a complex and stimulate viral IRES-mediated translation in the cytoplasm. This would be the case of PCBP2 (hnRNPE2) and SRp20 that relocalize together from the nucleus to the cytoplasm and synergistically stimulate PV IRES activity [232,248,249]. Other examples of nuclear proteins that, upon picornaviral infection, relocalize to the cytoplasm where they act as ITAFs are Sam68 [242,361], hnRNP D [255,362,363], hnRNP A1 [360,364], Nucleolin [270,360,365], hnRNPC [360], hnRNP K [360], and La [360], among others, as reviewed in [351,352]; by relocalizing nuclear proteins that act as ITAFs to the cytoplasm, viruses can cope with the need of these factors for viral IRES function.

**Figure 4:**
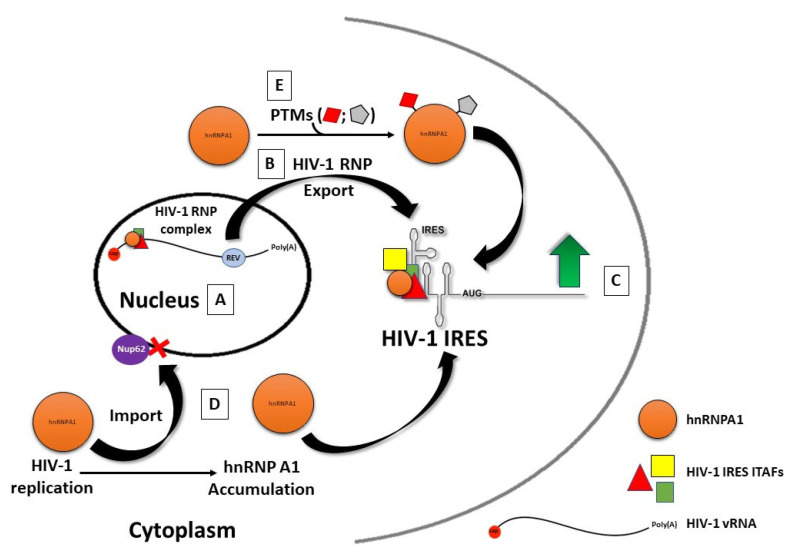
HnRNPA1: a model ITAF for the HIV-1 IRES. (**A**) In HIV-1 replicating cells, hnRNPA1 associates with the HIV-1 vRNA in the nucleus as part of the HIV-1 RNP [212,367]. (**B**) The HIV-1 RNP export is mediated by the viral protein Rev (reviewed in [367,373,374]). (**C**) HnRNPA1 stimulates (green arrow) HIV-1 IRES-mediated translation initiation [212,225]. (**D**) In cells, HIV-1 replication increases hnRNPA1 expression [212]. Cytoplasmic hnRNP A1 is re-imported into the nucleus by bound Trn-1, which interacts with the core member of the nuclear pore complex Nup62. However, in cells replicating HIV-1, Nup62 is downregulated, leading to the cytoplasmic retention of hnRNPA1. Cytoplasmic accumulation of hnRNPA1 further increases HIV-1 IRES activity [212]. (**E**) Post-translational modifications (PTMs) of hnRNPA1, including Mnk-mediated phosphorylation and PRMT5-induced symmetrical di-methylation of arginine residues, further promote HIV-1 IRES activity [212,225].

Retroviruses might use a different strategy to load some RBP onto their vRNA (Figure 4). Similarly to cellular mRNA, the retroviral vRNA assembles an mRNP during transcription [366,367]. This retroviral mRNP is dynamic in composition and, at later stages of replication, halts vRNA splicing, allowing the maintenance of an unspliced full-length vRNA. The full-length vRNA will serve two purposes; as a vRNA, it encodes the main viral polyproteins, and as the genomic RNA (gRNA), it will be incorporated into the newly formed viral particles [366,367]. It is noteworthy that some retroviruses synthesize one pool of gRNA that serves both purposes, while others synthesize two independent pools, one for translation and one for encapsidation [366,368,369]. In some retroviruses, translation of the vRNA can be mediated by an IRES [67,68]. Retroviral IRESs require ITAFs to function, several of which correspond to proteins preferentially located to the nucleus [68,198]. One example is hnRNPA1 [162,212,225], one of the most abundant nuclear proteins [370], which is primarily localized to the nucleoplasm, but also shuttles between the nucleus and the cytoplasm (Figure 4) [344]. In HIV-1 infected cells, hnRNP A1 binds the vRNA in the nucleus and is shuttled to the cytoplasm as part of the HIV-1 mRNP [212], later promoting HIV-1 IRES activity (Figure 4) [153,201,351,353]. HIV-1 replication imposes a blockade to the nuclear import of hnRNP A1, leading to an accumulation of the protein in the cytoplasm, further increasing HIV-1 IRES activity (Figure 4) [212]. It remains unclear if all ITAFs required for the HIV-1 IRES to function are loaded on the vRNA in the nucleus, as described for hnRNP A1 [212]. Some can probably also be loaded onto the mRNP complex in the cytoplasm [68,198,212].

It is noteworthy that, in the case of RBPs, their availability alone is not necessarily responsible for IRES function, as accessibility is also important [81,371]. Furthermore, in many cases, the protein binds to the RNA and other protein partners to promote or restrict IRES activity [81]. However, the cellular environment can regulate the protein’s ability to bind the RNA or interact with other protein partners [81]. For example, intracellular iron concentration alters the HCV 5′ UTR RNA protein binding pattern, impacting HCV IRES activity [372]. These observations suggest that IRES activity not only has an on/off function but can also be fine-tuned by changes in the cellular environment (Figure 3) [68,81].

## 8. Impact of Post-Translational Modification of ITAFs on IRES Activity

The mRNP code of any particular mRNA, including vRNAs, is not fixed and can be fine-tuned through PTMs of its associated proteins (Figure 3) [375,376,377,378,379,380,381,382,383]. Cells perceive and respond to their environment by regulating biological functions through, among other mechanisms, the induction of PTMs over specific target proteins [384]. PTMs, such as serine and threonine phosphorylation, arginines methylation, SUMOylation, and ubiquitination, alter RBP function by affecting their cellular localization, conformation, stability, and their ability to bind RNA or contact other proteins’ also part of the mRNP complex; this can result, for example, in a modified rate of mRNA translation [375,376,377,378,379,380,381,382,383]. Many viruses hijack the host’s PTM machinery to promote replication (reviewed in [375]). As mentioned above, FBP-2 inhibits EV71 IRES activity by competing and displacing ITAFs that promote IRES activity from the vRNA [251,385]. FBP-2 ubiquitination improves the competitive RNA binding ability of the protein, allowing it to displace other ITAFs from the RNA, thus enhancing its inhibitory effect on the EV71 IRES [385]. The RBP eIF5A is an ITAF for retroviral IRESs [295,296,386]. Activation of eIF5A by hypusination is required for this ITAF to promote the activity of the HIV-1 IRES, human T-cell leukemia virus type 1 (HTLV-1, *Retroviridae*, genus *Deltaretrovirus*) IRES, HTLV-1 basic leucine zipper protein (HBZ) IRES, and the MMTV IRES [295,386]. The inhibition of eIF5A-hypusination reduces the activity of these retroviral IRESs [295,386], suggesting that the PTMs of ITAFs directly impact retroviral IRES activity. The impact of PTMs on the ITAF function of RBP, such as hnRNPA1, has been extensively studied in the context of cellular IRESs [81,378,379,380,381,382,383]. Some reports have also addressed the impact of hnRNP A1 and hnRNPA1-PTMs on retroviral IRESs [162,212,225,387]. Reports show that the mitogen-activated protein kinase (MAPK)-interacting protein kinase (Mnk)-mediated phosphorylation of hnRNP A1 contributes to HIV-1 IRES activity (Figure 4) [212]. The treatment of cells with inhibitors of Mnk activity selectively reduced HIV-1 IRES activity without impacting cap-dependent translation initiation or hnRNP A1 cytoplasmic localization [212] (Figure 4). Another example is the arginine methyltransferase 5 (PRMT5)-induced symmetrical di-methylation of the arginine (SDMA) residues of hnRNP A1, which is required for the ITAF to promote HIV-1 and HTLV-1 IRES activity [225] (Figure 4). Interestingly, the same PRMT5-induced SDMA of hnRNPA1 inhibits MMTV IRES activity [225]. Thus, PTMs might significantly alter ITAF function, deferentially impacting the activity of viral IRESs.

## 9. ITAFs and IRES-Dependent Viral Tropism

The primary determinant of viral cell tropism is the availability of the viral receptor on the cell surface [388]. However, upon entry, viral processes such as IRES-mediated vRNA translation also require host factors. The availability of ITAFs varies according to cell type and depending on the cell’s physiological state [80,81,371,389]. For example, although widely expressed, hnRNPs vary in concentration between different tissues [349]. For this reason, the cell tropism and viral pathogenesis might rely not only on the ability of the virus to enter a particular cell type but also on the compatibility of cis-acting viral regulatory elements such as the IRES and the host cell repertoire of ITAFs [125,157,188,390,391,392]. IRES-dependent viral tropism implies that different subsets of ITAFs are expressed or available for IRES-activity in a cell-type-specific manner [125,157,188,390,391,392]. For example, TMEV neurovirulence strongly relies on its IRES function, which is highly dependent on PTB, yet the ITAF is deficient in neural cells [393]. Neural cells do express nPTB, which is unable to replace PTB as an ITAF for the TMEV IRES, linking ITAF availability with the attenuation of TMEV neurovirulence [393]. This observation directly links cell-specific ITAFs with virus pathogenicity [125,157,188,390,391]. Further confirming this observation are assays showing that replacement of the IRES of TMEV by the IRES of FMDV—the activity of which is dependent on PTB and Ebp1 [234]— in the context of the TMEV vRNA yielded viable but completely attenuated viruses that fail to replicate in mouse neurons [234]. Another example is the attenuation of PV-Sabin3 IRES in the central nervous system (CNS) [394]. The PV-Sabin3 IRES harbors a mutation in one of the PTB binding sites within the PV IRES, which alters the RNA’s secondary structure leading to reduced binding of PTB to the PV-Sabin3 IRES [394]. Interestingly, a PV-Sabin3 IRES translation defect in CNS was rescued by overexpressing PTB, but not nPTB [394], showing that the brain-specific PTB paralog could not replace the ITAF function of PTB. Together, data suggested that PV-Sabin3 CNS attenuation was due to the low levels of PTB within the CNS and its reduced binding to the IRES [394]. Thus, tissue-specific levels of an ITAF can severely impact the pathogenesis of viruses that exclusively rely on IRES-mediated translation initiation for their protein synthesis [234,393,394].

As viral fitness partially depends on IRES function, it is not unexpected that adaptive mutation within the IRES may appear and be fixed in the vRNA during virus replication to improve vRNA translation. Most RNA viruses circulate as quasispecies; this is mainly due to the “error-prone nature” of viral RNA-dependent RNA polymerases or their reverse transcriptases in the case of retroviruses [395,396]. In this scenario, it is not unexpected to identify viral variants that, when compared, exhibit nucleotide variations within the RNA region that harbors their IRES activity. Interestingly, some nucleotide variations are silent regarding the vRNA translation rate, while others impact IRES activity. An example corresponds to the HAV IRES. The HAV IRES is weak when compared to other picornaviral IRESs [142,188]. The weak activity of the HAV IRES is attributed to a lower affinity of the HAV 5′ UTR for translation initiation factors and the low abundance of ITAFs required for its optimal activity in several cell lines [48,142]. However, cell culture-adaptive mutations within the HAV IRES contribute to enhanced replication and cell-type-specific translation efficiency [397,398]. Several cell culture adaptive mutations within the HCV IRES have also been reported [399,400,401,402,403,404,405,406]. A list of reported HCV IRES variants can be found in the HCVIV database [407]. Similar findings have been described for the HIV-1 vRNA [162,408]. However, the molecular mechanisms by which the identified nucleotide variation alters HIV-1 IRES activity remain unknown [162,408].

A feature first described for picornaviruses is that their IRESs are partially and even totally exchangeable, allowing the assembly of replication-competent chimeric viruses [409,410,411]. For example, the PV IRES was replaced by the HCV IRES generating a PV/HCV chimeric virus [412]. Chimeric IRESs have also been made between the HCV/CSFV [413]. A general result of these experiments is that the translational properties and cell-specific protein synthesis of the chimeric viruses were determined by the IRES and not by the rest of the viral sequences included in the constructs [173,392,410,411,414,415,416], strengthening the notion of IRES-dependent viral tropism. An example is a chimeric virus where the cognate PV IRES was replaced with the HRV2 IRES that grows with wt kinetics in HeLa and malignant glioma cells, but is severely attenuated for growth in neuron-like cell lines [410,417].

Cell-type-dependency of IRES activity has also been observed for the IRESs from other viral families. For example, the HIV-1 IRES is most active in T-cell and monocyte cell lines, which are derived from the natural cellular targets of HIV-1 when compared to other cell lines [125]. This observation was also attributed to the uneven distribution of ITAFs in different cell lines [125].

The need for specific ITAFs and their uneven distribution among different cell types might partially explain why the function of some viral IRESs is cell-type-dependent [125,188,349,416,418]. The dependency on specific ITAFs would also restrict cross-kingdoms IRES activity. For example, the HCV IRES is functional in mammalian cells but not insect cells [188,419]. Similar findings have recently been reported for the DENV IRES, which also exhibit poor activity in insect cells [66,189]. Among the *Picornaviridae* IRESs, which show cell-type restriction, an exception would be the EMCV IRES, which has been reported to be moderately active in plants cells [420]. A few viral IRESs, however, function in multiple cell types. A common feature of these broad-cell type IRESs, some of which can even cross kingdoms (at least two kingdoms), is that they have null or minimal requirements of ITAFs to function [104,105,421,422]. For example, the crTMV IRES is active in RRL and wheat germ extract (WGE) in-vitro translation systems [423]. The RhPV 5′ IRES functions efficiently in mammal-, insect-, and plant- cell-free in-vitro translation systems and can form 48S initiation complexes invitro with only the mammalian initiation factors eIF2, eIF3, and eIF1 [105,166]. Other examples are the CPV IGR IRES and the *Plautia stali* intestine virus (PSIV; *Dicistroviridae*, genus *Triatovirus*) IGR IRES, which are functional in mammalian, insect, and plant cell-free in-vitro translation systems [104,106,421,424]. The CPV IGR IRES is also functional in yeast [421]. The PSIV IGR IRES exhibits an even more remarkable feature, also being active in prokaryotes, directly challenging the paradigm that prokaryotic and eukaryotic translation initiation mechanisms are mutually exclusive [422]. RNA structure integrity was shown to be essential for PSIV IGR IRES-mediated translation in bacteria [422], suggesting an RNA-structure-based signal capable of hijacking both the eukaryotic and prokaryotic translational machinery. It is noteworthy that despite being functional in different cell types from various kingdoms [104,105,421,422], the translational activity of a particular IGR IRES may greatly vary from one specific cell type to another [111,419]. Furthermore, it is important to consider that not all *Dicistroviridae* IRESs share the ability to be active in all cellular backgrounds. For example, the CrPV 5′ IRES does not function efficiently in plant-cell-free in-vitro translation systems [104], and the 5′ IRES of the PSIV vRNA is active only in insect cell lysates, not in mammals or plant-cell-free in-vitro translation systems [106].

## 10. What about Cellular IRESs?

As mentioned above, about 10–15% of all cellular mRNAs harbor IRESs [57,79,82,87]. Cellular IRESs have been extensively reviewed [11,14,79,80,82,87,389,425,426,427]. Even though cellular and viral IRESs share the same function of recruiting the 40S ribosomal subunit internally, they strongly differ in their specific features [56,389,428,429]. In general, cellular IRESs are less structured than viral IRESs. Indeed, some cellular IRESs completely lack a secondary structure [430]. Furthermore, and in sharp contrast to viral IRESs, nucleotide deletions within cellular IRESs rarely disable translation [430,431,432,433], as individual sections of the IRESs can promote internal initiation, albeit not as efficiently as the entire element [431,432,433]. Cellular IRESs are disabled by mutations or deletions that disrupt more than one structural region [434]. So, in contrast to most viral IRES, which are viewed as a unique structural RNA element where single point mutations within the IRES can significantly alter its function [95,99,101,144,156], cellular IRESs are composed of multiple short modules. The combined effect of these modules promotes internal initiation [431,432,433,435]. An additional feature of cellular IRESs is their high dependency on eIFs and ITAFs [81,82,223,389,428]. ITAFs associate with the mRNA to conform a translationally active RNP complex, the IRESome (Figure 2 and Figure 3B) [81]. In addition, cellular IRESs tend to be active only when the mRNA is synthesized in the nucleus and do not function in direct RNA transfection assays or in DNA transfections where mRNA is synthesized in the cytoplasm (T7 RNA polymerase dependent transcription) [436,437,438]. One possible explanation for the need for this “nuclear experience” is that the IRESome assembly initiates in the nucleus during RNA polymerase II-driven transcription. Thus, translation from IRES-containing cellular mRNAs is cap-dependent under normal physiological conditions, as the IRES is “OFF” (Figure 3). However, under stress or other physiological conditions such as apoptosis, when cap-dependent translation is repressed, ITAFs associate with the needed mRNAs, and translation is re-programmed as their IRESs are turned “ON” [81,389,427,428]. Therefore, cellular IRESs are switched on during physiological and pathophysiological conditions that inhibit cap-dependent translation initiation [80,81,223,389,427]. Thus, in general, in a normal cell, IRESs represent a survival switch required to drive the synthesis proteins that play a role in cell maintenance under conditions that repress cap-dependent translation [80,81,389,427]. Furthermore, cellular IRESs have been shown to accomplish relevant physiological functions, for example, controlling gene expression during development [79,81]. However—and most probably due to variation in the concentration availability of specific ITAFs or combination of ITAFs—in different cell types, cellular IRESs are not equally functional in different cellular backgrounds [81,371,389,428,438], suggesting a cell-type-dependent translational control of IRES activity. It is noteworthy that the interest in understanding the mechanisms of the function of cellular IRESs has been recently boosted by reports showing that some cellular endogenously generated cccRNAs are translated [439,440,441]. This is because, in eukaryotic cells, cccRNAs require IRESs to initiate translation.

## 11. Concluding Remarks

The diversity of RNA structures, or RNP-complexes, that different viral RNAs have evolved to enable internal initiation to outcompete the host’s mRNAs during infection is remarkable. For many viral IRESs, there are still many unknowns regarding the molecular mechanisms driving their function. Fine-tuning of IRES activity during viral replication also emerges as a novel and exciting question. As IRESs might not be isolated players that turn on to enable viral protein synthesis, but are part of a more complex network of signals that sense the cellular environment regulating viral gene expression accordingly during infection. This would imply that ITAFs play an essential role in fine-tuning viral IRES function through their localization (nucleus/cytoplasm), binding affinity, and capacity to interact with other regulatory proteins. The understanding of how the PTMs of ITAFs impact viral IRES activity also emerges as a fascinating research area. Furthermore, little is known on how viral and cellular-induced PTMs of ITAFs are important for timing viral gene expression during replication, or their relevance in virus-induced pathogenesis.

## Figures and Tables

**Table 1:** Summary of most studied ITAFs for viral IRESs.

ITAF	Viral IRES (Type of Modulation)	Binding Region	Main RNA Binding Domain (RBD)	Domain Description/Structure	Type of RNA that is Bound
hnRNPI (PTB)	Picornavirus EMCV (promotes) [259]	IRES [259]	RNA-recognition motif (RRM)	RRM is involved in different processes of gene expression, it has two consensus sequences RNP1 (Lys/Arg-Gly-Phe/Tyr-Gly/Ala-Phe/Tyr-Val/Ile/ Leu-X-Phe/Tyr), and RNP2 (Ile/Val/Leu-Phe/Tyr-Ile/Val/Leu-XAsn-Leu), where X can be any amino acid [260,261]. The RRM has an αβ sandwich fold. The RNP1 and RNP2 motifs are positioned in the central strands of the β sheet [260,261].	ssRNA [261,262].
	Picornavirus EV71 (promotes) [263]	5′ UTR [263]			
	Picornavirus PV (promotes) [264]	IRES (Domain V) [264]			
	Picornavirus HRV (promotes) [250]	IRES [174]			
	Picornavirus HAV (promotes) [265]	5′ UTR [265]			
	Picornavirus CVB3 (promotes) [266]	3′ and 5′ UTR [266]			
	Flavivirus HCV (promotes) [267]	IRES [267]			
	Retrovirus MMTV (promotes) [268]	5′ UTR [268]			
Nucleolin	Picornavirus PV(promotes) [269]	IRES [269]			
	Picornavirus HRV (promotes) [269]	Unknown			
	Picornavirus FMDV (promotes) [270]	Unknown			
hnRNPD (AUF1)	Picornavirus PV, HRV (reduces) [253]	5′ UTR [253]			
	Picornavirus EV71 (reduces) [254]	5′ UTR (stem loop II) [254]			
	Flavivirus HCV (promotes) [271]	5′ UTR [271]			
La	Picornavirus PV (promotes) [272]	5′ UTR [272]			
	Picornavirus EMCV (promotes) [230]	IRES [230]			
	Picornavirus CVB3 (promotes) [273]	3′ and 5′ UTR [273]			
	Picornavirus HAV (reduces) [274]	5′ UTR [274]			
	Flavivirus HCV (promotes) [272]	5′ UTR [272]			
PSF	Picornavirus CVB3 (promotes) [240]	IRES [240]			
SRp20	Picornavirus PV (promotes) [248,249]	stem-loop IV [248,249]			
hnRNP L	Flavivirus HCV (promotes) [275]	IRES [275]			
hnRNP Q	Flavivirus HCV (promotes) [276,277]	downstream of the initiation codon [276,277]			
hnRNP C1/C2	Picornavirus CVB3 (reduces) [278]	IRES (Stem-Loop V) [278]			
hnRNP K	Picornavirus EV71 (promotes) [279]	5′ UTR (cloverleaf) [279]	K-homology domain (KH)	Involved in gene expression processes. Comprises about 70 residues with a hydrophobic cleft, formed by a Gly-X-X-Gly segment and a variable loop. KH domains are present in multiples copies [262,280,281]. Two or three alpha-helices clustered on the surface of antiparallel beta-sheets [262].	ssRNA [262].
	Flavivirus HCV (promotes) [282]	5′ UTR [282]			
	Picornavirus FMDV (reduces) [256]	IRES (II, III and IV domains) [256]			
FBP-2	Picornavirus EV71 (reduces) [251]	IRES [251]			
hnRNPE1(PCBP1)	Picornavirus EV71 (promotes) [283]	5′ UTR [283]			
	Picornavirus PV (promotes) [284]	5′ UTR (cloverleaf) [284]			
	Picornavirus HRV (promotes) [284]	IRES [284]			
hnRNPE2(PCBP2)	Picornavirus PV (promotes) [232,233,284]	5′ UTR (cloverleaf) [284]			
	Picornavirus HRV (promotes) [284]	IRES [284]			
	Picornavirus HAV (promotes) [285,286]	5′ UTR (pyrimidine-rich tract) [285,286]			
	Flavivirus HCV (promotes) [284]	IRES [284]			
Sam68	Picornavirus EV71 (promotes) [239]	5′ UTR (Stem-loops IV and V) [239]			
Staufen 1	Flavivirus HCV (promotes) [287]	3′UTR (variable-stem-loop region) and IRES (domain IIId) [287]	Double-stranded RBD (dsRBD)	Domain of 70–90 amino acids, involved in RNA maturation [262,288,289,290]. Conserved topology α1-L1-β1-L2-β2-L3-b3-L4-α2, where L specifies a loop [262]	dsRNA [262,288,289,290].
	Picornavirus EV71 (promotes) [291]	5′ UTR [291]			
	Retrovirus HIV-1 (promotes) [292]	5′ UTR [292]			
HuR	Picornavirus EV71 (promotes) [241]	Stem-loop II [241]			
	Flavivirus HCV (promotes) [224,293]	Unknown			
	Retrovirus HIV-1 (reduces) [224]	Does not bind the IRES [224]			
Gemin 5	Picornavirus FMDV (reduces) [257]	IRES [257]			
	Flavivirus HCV (reduces) [257]	IRES [257]			
DRBP76	Picornavirus HRV 2 (reduces in neuronal cells) [258]	IRES (sldV/VI) [258]			
Ebp1	Picornavirus FMDV (promotes) [234]	IRES (Domain I) [234]			
Unr	Picornavirus HRV (promotes) [237]	5′ UTR [236]	Cold shock domain (CSD)	CSD is part of catalytic centers by binding different biomolecules. Participates in various processes of gene expression regulation [294]. Five-stranded antiparallel beta-barrel structure [294]	ssRNA [294]
	Picornavirus PV (promotes) [237]	Unknown			
	Picornavirus HRV-2 (promotes) [238]	5′ UTR [238]			
eIF5A	Retrovirus (HIV-1, HTLV-1, MMTV) (promotes) [295,296]	Unknown			
hnRNP A1	Retrovirus HIV-1 (promotes) [213]	5′ UTR [225]	RGG or GAR boxes	RBPs containing these repeats function in RNA metabolism processes such as transcription, splicing of pre-mRNA, localization, and post-translational modification [297]. Gly-rich regions are interspersed with aromatic and Arg residues and are present in disorder protein regions [298]	G-quadruplexes [299]
	Retrovirus HTLV-1 (promotes) [225]	Unknown			
	Retrovirus MMTV (promotes) [225]	Unknown			
	Picornavirus EV71 (promotes) [300]	Stem-loop II and VI [300]			
Ago2	Picornavirus EV71 (promotes) [241]	EV71 IRES (stem-loop II) [241].	P-element-induced whimpy testes (PIWI) domain, a middle (MID) domain, and a PIWI/Argonaute/Zwille (PAZ) domain [301].	Required in gene silencing by the RNA-induced silencing complex (RISC) [301,302]. Bi-lobed architecture composed of four globular domains (N-terminal(N), PIWI, MID, and PAZ connected through two structured linker domains [301].	ssRNA [301,302].
	Flavivirus HCV (promotes) [303]	Stabilizes mir-122/HCV RNA interaction [303,304].			
HSPA6	Picornavirus EV-A71 (promotes) [245]	Unknown	Nucleotide-binding domain (NBD), also known as the ATPase domain.	Found in Hsp70 family proteins with ATP-regulated chaperone function [305]. Two lobes connected by α-helix in C-terminal [305].	unknown
HSPA8	Picornavirus EV-A71 (promotes) [243]	Does not bind to the 5′-UTR [243]			
HSPA1 and HSPA9	Picornavirus EV-A71 (promotes) [244]	Unknown			

## Data Availability

Not applicable.
